# Bioinformatics analysis of the epitope regions for norovirus capsid protein

**DOI:** 10.1186/1471-2105-14-S4-S5

**Published:** 2013-03-08

**Authors:** Liping Chen, Di Wu, Lei Ji, Xiaofang Wu, Deshun Xu, Zhiwei Cao, Jiankang Han

**Affiliations:** 1 Huzhou Center For Didsease Control and Preventron, Zhejiang 311000, China; 2 School of Life Sciences and Technology, Tongji University, Shanghai 200092, China; 3 Shanghai Center for Bioinformation Technology, Shanghai 200235, China

## Abstract

**Background:**

Norovirus is the major cause of nonbacterial epidemic gastroenteritis, being highly prevalent in both developing and developed countries. Despite of the available monoclonal antibodies (MAbs) for different sub-genogroups, a comprehensive epitope analysis based on various bioinformatics technology is highly desired for future potential antibody development in clinical diagonosis and treatment.

**Methods:**

A total of 18 full-length human norovirus capsid protein sequences were downloaded from GenBank. Protein modeling was performed with program Modeller 9.9. The modeled 3D structures of capsid protein of norovirus were submitted to the protein antigen spatial epitope prediction webserver (SEPPA) for predicting the possible spatial epitopes with the default threshold. The results were processed using the Biosoftware.

**Results:**

Compared with GI, we found that the GII genogroup had four deletions and two special insertions in the VP1 region. The predicted conformational epitope regions mainly concentrated on N-terminal (1~96), Middle Part (298~305, 355~375) and C-terminal (560~570). We find two common epitope regions on sequences for GI and GII genogroup, and also found an exclusive epitope region for GII genogroup.

**Conclusions:**

The predicted conformational epitope regions of norovirus VP1 mainly concentrated on N-terminal, Middle Part and C-terminal. We find two common epitope regions on sequences for GI and GII genogroup, and also found an exclusive epitope region for GII genogroup. The overlapping with experimental epitopes indicates the important role of latest computational technologies. With the fast development of computational immunology tools, the bioinformatics pipeline will be more and more critical to vaccine design.

## Background

Norovirus is a category of small non-enveloped icosahedral viruses from *Caliciviridae *family with diameter of ~38 nm. Despite of the low mortality, approximately 50% of all gastroenteritis outbreaks have been reported to be caused by norovirus[1]. Actually it is the major cause of nonbacterial epidemic gastroenteritis in both developing and developed countries [2], since being firstly described in 1968 during an outbreak in an elementary school in Ohio[3]. Fast diagnosis and treatment is critically needed in clinical cases. Genetically, norovirus have been classified into five genogroups according to the difference of capsid protein sequnces (genogroup I [GI] to genogroup V [GV]). Among the five of them, only GI and GII types can infect human to cause norovirus outbreak cases in community. 25 different sub-genotypes have been further identified for GI and GII [4]. Sub-genogroup of GII.4 has been frequently detected as the major pathogen for most reported cases [5].

The genome of norovirus involves a ~7.5 kb positive-sense, single-stranded RNA with three open reading frames (ORF1~ORF3) [6]. ORF1 is over 5 kb and occupies the first 2/3 of the genome. A 200 kDa polyprotein was encoded by ORF1 which can be autoprocessed by a virally encoded protease to yield the non-structural viral replicase proteins essential for viral replication. Then ORF3 encodes a 22 kDa small basic structural protein possibly packaging the genome into virions [7]. At last, ORF2 encodes the major capsid protein VP1, 57 kDa, also believed to be the major antigen protein for the virus. VP1 protein includes the shell (S) domain which is highly conserved among different noroviruses and the protruding (P) domain with N-terminal P1, C-terminal P1, and P2 parts. The P2 domain was reported to be the most protruding and diverse among different norovirus groups [8], indicating its critical function in interacting with host.

Due to the lack of a suitable cell culture system or animal model, the study of norovirus was greatly hampered initially. But recently a significant advance has been achieved by using virus-like particles with the expression of the viral capsid protein in the baculovirus expression system [9]. With this method, the capsid protein of norovirus can be expressed in an *Escherichia coli *system with the immunological resembling to the native capsid protein.

To differentiate the many sub-groups of virus quickly, several monoclonal antibodies (MAbs) have been developed based on *E. coli*-expressed norovirus capsid proteins [10].

Although most of the binding epitopes recognized by MAbs for norovirus were reported to be located conservatively in the C-terminal P1 domain, different binding characteristics have been reported for these MAbs in previous research works [11-13]. One study showed that a MAb14-1 could recognize 15 recombinant virus-like particles (GI.1, 4, 8, and 11 and GII.1 to 7 and 12 to 15) and show the broadest recognition range of any existing MAb to norovirus proteins [11]. The binding sites were at the C-terminal P1 domain of VP1 protein (amino acid positions 418 to 426 and 526 to 534). In another study, 10 strains of noroviruses (4 in GI and 6 in GII) were recognized by a group of MAb obtained from orally-immunized mice [14]. Also there were MAbs whose binding sites are besides the C-terminal P1 domain. In one study a cross-reactive MAb between human GI and bovine GIII was reported [15]. Recently, a MAb N2C3 recognizing genogroups I, II, III and V was reported [16]. This is the first to report a cross-reactive monoclonal antibody which is able to detect both human and animal-associated norovirus. The binding site of N2C3 was in the in the beginning section of VP1 ^55^WIRNNF^60^.

From the above reviews, it can be seen that some antibody can just recognize one specific sub-genogroup of norovirus, and some own the multi-recognition activity for several sub-genogroups of norovirus. There is still no antibody seen which can recognize all the human infected strains, like all GIs and GIIs. On the other hand, the previously reported epitope mainly focuses on the C-terminal P1 domain, but there are indications that the N-terminal of VP1 may also be important area to induce antibody binding. Are these epitope areas closely related? We need to investigate their structural or conformational epitopes. Considering that the virus is keeping mutating and the epitope area at VP1 protein might change significantly especially in the 3D structure so that the known antibody may no more be able to recognize, it is necessary to systematically study the various features of all known VP1 proteins of norovirus sequences, especially those human infected groups (GI and GII groups). However, there is no report being seen to investigate the similarity and difference between the epitope regions of different sub-genogroups of norovirus. In the mean time, a comprehensive and comparative analysis for the epitope regions can be made between diverse sub-genogroups, with the development of bioinformatics technolgy. Such work may provide hints to formulate future antibodies targeting one or overall sub-genogroups of norovirus.

In this study, we collected the VP1 sequences of norovirus for GI and GII sub-genogroups. With homology modeling, the 3D structures of these noroviruses have been generated. According to the modeling 3D structures, SEPPA was used to predict the potential epitope regions. Combining with the previous reports of MAbs for the norovirus, the binding character was discussed among diverse noroviruses.

## Methods

### Sequences and sequence analysis

A total of 18 full-length human norovirus capsid protein sequences were downloaded from NCBI, including 7 GI (ABW74128, ACN32270, AAS86780, ACU56258, ACX33982, ACV41096, ADB54834) and 11 GII (AAL13016, BAG68716, ADK23787, AEG79292, ABC96332, ABL74397, ABL74391, ADE28721, ACX85810, ADZ24003, ACX81355). The genotypes include GI.1, 2, 3, 4, 8 and GII.1, 2, 3, 4, 6, 7, 12, 13. The homology of the sequences and phylogenetic tree was constructed using MEGA3.1 [17].

### Protein 3D structure modeling and conformational epitope prediction

The 3D structures were modeled for 7 GI and 11 GII norovirus VP1 proteins. From the PDB database, we get the 3D structures of GI.1, GII.4 and Murine norovirus 1 as the template structures. Protein modeling was performed with program Modeller 9.9 and default parameters (http://www.salilab.org/modeller/9.9/release.html, [18]). The modeled 3D structures of capsid protein of norovirus were submitted to the protein antigen spatial epitope prediction webserver (SEPPA) for predicting the possible spatial epitopes with the default threshold [19]. SEPPA (Spatial Epitope Prediction of Protein Antigens) server is a tool for conformational B-cell epitope prediction. With 3D protein structure as input, each residue in the query protein will be given a score according to its neighborhood residues' information. The predicted epitope regions were mapped to the protein sequences of VP1, and the ClustalX software (version 1.83) [20] was further used to make a multiple sequence alignment for the epitope regions of various subgenotype of noroviruses. The results were processed using the BioEdit software (version 7.0.5.2;) [21].

## Results

### Sequence similarity of noroviruses for different sub-genogroups

The 18 full-length protein sequences of noroviruses capsid proteins have been downloaded from NCBI. With the sequence alignment, we detected the sequence similarity between these sub-genogroups capsid proteins. According to the sequence similarity, GI sub-genotype and GII sub-genotype were clustered to the divided branches in the phylogenetic tree analysis. The sequence similarity for 7 GI-type sequences was 64.5%-100%, and the similarity for 11 GII-type sequences was 63.1%-95.2%. The similarity between GI and GII sub-genogroup is about 40.9%-47.4% (results are not shown). The similarity between sub-genogroups is expectedly lower than the sequence similarity among each genogroups. According to the sequence alignment results, the sequence mutations mainly distribute in P2 domian for both GI and GII, which is agreeing with previous reports.

**Figure 1:**
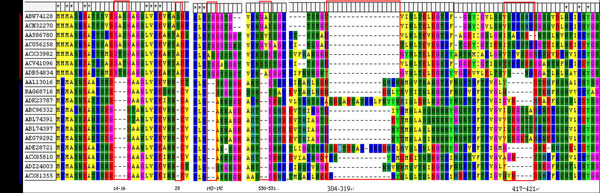
**Four sites deletion of GII and unique insertion of GII.3, GII.6 and GII.4**. Four sites deletion of GII (14-16\28\192-193\530-531); unique insertion of GII.3 and GII.6 (304-319); unique inserted fragment of GII.4 at 417-421.

Compared with GI, we found that the GII genogroup had four deletions in the VP1 region, including, ^14^GAS^16 ^and A^28 ^in the N-terminal domain, ^192^GS^193 ^in the S-domain and ^530^GA^531 ^in the C-terminal P1 domain, respectively. At the same time, exclusive insertion segments have also been observed on some sub-genotypes of GII. GII.3 and GII.6 both had a 16 amino acid insertion fragment at 304-319 position in sequence, and the sequences of insertion segments are different with each other for GII.3 and GII.6. As the most prevalent genetic cluster of norovirus, the sequence of GII.4 has also a special segment of a 5~6 amino acid insertion at 417-421 position in sequence which have not been observed for other sub-genotypes in GII genogroups. For the GI genogroup, we have not observed the insertion in sequences. Depending on the sequence alignments, the sequences of capsid proteins show the obvious difference between GI and GII. For sub-genogroups of GII.3, GII.4, and GII.6, there are the exclusive insertion fragments, as can be seen in Figure 1.

### 3D structures of norovirus capsid protein

The 3D structures of 21 norovirus capsid proteins were selected as templates for homology modeling, including 3Q39, 3Q3A, 3Q6Q, 3Q6R, 3R6J, 3R6KA, 2ZL6, 3ONU, 3LQ6, 3M81, 2GH8, 2OBS, 3ONY, 3PA1, 3PA2, 3SEJ, 1IHM, 3PUM, 3PUN, 3PVD, 3Q38. With these templates, the 3D structures of 18 representative capsid proteins have been homologically modeled.

**Figure 2:**
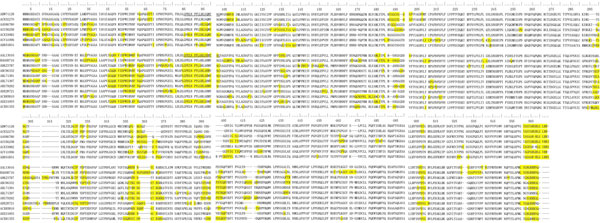
**The result of sequential alignment for norovirus capsid proteins**. The result of sequential alignment for norovirus capsid proteins, the potential spatial epitope are highlighted with yellow. The above 7 sequences were GI, and the below 11 sequences were GII.

**Figure 3:**
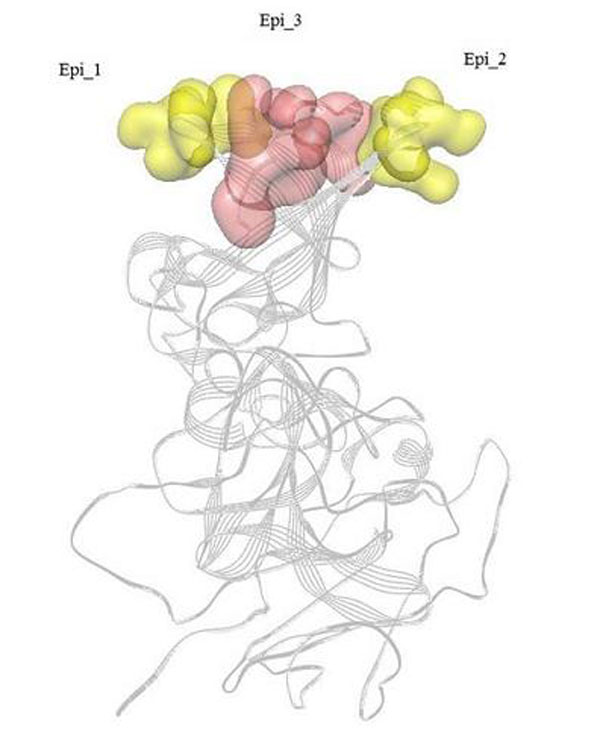
**Two common epitope regions for GI and GII genogroup and the exclusive epitope region for GII genogroup**. The spatial epitope of norovirus capsid protein. The 3D structure of norovirus capsid protein is displayed as the ribbon mode, and the common and exclusive spatial epitopes for GI and GII have been mapped to the 3D structure. Two common epitope regions (Epi 1: 298~305, Epi 2: 357~374) for GI and GII genogroup have been highlighted in yellow color and surface mode. The exclusive epitope region for GII genogroup (Epi_3: 395~406) has been highlighted with red color.

The modeled structures of VP1 protein were submitted to SEPPA to detect the potential conformational epitope positions. The prediction results were summarized in Figure 2, and the residues in conformational epitope regions have been highlighted with yellow. As we can see from the results, the predicted conformational epitope regions mainly concentrated on N-terminal (1~96), Middle Part (298~305, 355~375) and C-terminal (560~570). With consideration of the flexibility for protein structures at the N-terminal and C-terminal regions, we focused our analysis on the middle part. In general, the positions of potential conformational epitope regions on sequences are similar or adjacent to each other for GI and GII genogroups. We can find two common epitope regions on sequences (Epi 1: 298~305, Epi 2: 357~374) for GI and GII genogroup, and also found an exclusive epitope region for GII genogroup (Epi_3: 395~406). We have mapped the conformational epitope regions to the Figure 3.

## Conclusions

As the protective mechanism for human beings, immune system is implicated in recognizing and defending the foreign antigens, where the adaptive immune system or antibody system is considered to be the dominate process. Specific antibodies are developed gradually by the B cell lymphocytes to specifically interact with and neutralize the corresponding antigens, while the recognition of antigens depends on a cluster of sites located on the antigen surface named the epitopes. Among the different types of antigens, the protein antigen is the top one which has been intensively investigated and accumulated so far. Analysis of protein epitopes has become increasingly hot because of the expectation to facilitate the design of monoclonal antibodies and even the novel vaccines especially at the current time of continuous outbreak of newly emerging diseases.

As to the norovirus, we have discussed the similarity and difference between the sequences and conformational epitopes of capsid proteins for GI and GII genogroups. With the comparison results, we found the exclusive insertions on sequence for GII.3, GII.4, and GII.6. As to these exclusive insertions, it is more interesting that their spatial positions on the VP1 protein are close to the epitope regions. The exclusive insertion for GII.3 and GII.6 sub-genogroups is close to the common epitope regions, and the exclusive insertion for GII.4 is close to exclusive epitope regions for GII genogroup in 3D structure. On the other side, more and more reported cases of norovirus have been confirmed to be sub-genotypes GII.4. As to the sub-genotype of GII.4, with the comparison of the potential conformational epitope, we also find that there are some special usage of amino acids on sequence, such as ^81^WSAP^84^, ^181^K, ^241^E and ^261^S for GII.4 and ^81^LNLE^84^, ^181^R, ^241^G/S and ^261^E for other GIIs. These residues are exposed to the solvent and near the epitope regions in 3D structures. It's hard to determine how such spatial distribution for these sequence insertion will contribute the specific antigenicity for these sub-genogroups norovirus without further investigation. But such insertion will affect recognition between antibody and epitopes undoubtedly.

Several MAbs have been reported to be used to detect different sub-genogroups of *E. coli*-expressed norovirus capsid proteins in clinical samples of norovirus infections [10]. Most of the binding epitopes recognized by MAbs for norovirus were located conservatively in the C-terminal P1 domain, and different binding characteristics have been reported for these MAbs in previous research works [11-13]. The MAb N2C3 can recognize the segment of ^56^WIRNNF^61 ^as the epitope regions for GI, GII, GIII and GV [22]. Another antibodies of 1B4 and 1F6 can recognize 87-103 for GI and GII [10,23]. These experimentally confirmed epitope regions partly overlapped with our predicted conformational epitope regions.

From our results, we also find that there are some common epitope regions with the similar chemical character and spatial position on the surface of capsid proteins of different sub-genogroups noroviruses. This may be the reason that some monoclonal antibody can recognize various noroviruses in genogroups I and II. In previous research, the recombinant virus-like particles (VLPs) have been wildly used as an immunogen to stimulate and prepare the monoclonal antibody [22-24]. VLPs is the end-product of a 58 kDa protein by the expression of norovirus capsid protein in the baculovirus translation system. Now, the comparison between sequence, structure and potential epitope regions remind us that we may find some common segments in the epitope regions of norovirus and testify the feasibility of this common segments as the linear peptide to stimulate the corresponding antibody with a binding affinity.

In this work, the epitope of capsid protein VP1 was in-silico investigated for norovirus at sequence and structural level with comparison to known experimental results and domain knowledge. The overlapping with experimental epitopes indicates the important role of latest computational technologies, while the novel finding may be helpful to future wet-lab design. It can be expected that, with the fast development of computational immunology tools, the bioinformatics pipeline will be more and more critical to vaccine design.

## Competing interests

The authors declare that they have no competing interests.

## Authors' contributions

Liping Chen, Lei Ji, Xiaofang Wu, Deshun Xu and Jiankang Han carried out the sequences analysis and the 3D structures modeling. Di Wu and Zhiwei Cao participated in the possible spatial epitopes predicting. Liping Chen drafted the manuscript. Di Wu helped to draft the manuscript. All authors read and approved the final manuscript.

## Declarations

The funding for publication of this article is provided by the Huzhou Science and Technology Bureau (2011C23064).

This article has been published as part of *BMC Bioinformatics *Volume 14 Supplement 4, 2013: Special Issue on Computational Vaccinology. The full contents of the supplement are available online at http://www.biomedcentral.com/bmcbioinformatics/supplements/14/S4
